# Supplementary material for: Cohort profile: Life-course experiences and pregnancy (LEAP)–A retrospective pregnancy cohort for life-course reproductive health research
Source: PLoS One. 2024 Mar 20;19(3):e0295825. doi: 10.1371/journal.pone.0295825 (PMC10954138; doi:10.1371/journal.pone.0295825)
Supplement: S1 File — (DOCX) [file pone.0295825.s001.docx]

**Table of Contents**

Survey Introduction

[A. Screening for Pregnancy and Births 3](#_Toc5696966)

[B. Pregnancy and postpartum health, about EACH LIVE BIRTH 4](#_Toc5696968)

[C. Pregnancy psychosocial and behavioral factors, about FIRST LIVE BIRTH 8](#_Toc5696969)

[D. Postpartum characteristics, about FIRST LIVE BIRTH 17](#_Toc5696970)

[E. Questions about Current Pregnancy (women indicating currently pregnant and may or may not have had a previous birth) 19](#_Toc5696971)

[F. Questions about infertility (women indicating no births and not currently pregnant) 29](#_Toc5696974)

[G. Current Health: Questions for Women within 12 months postpartum from most recent birth 30](#_Toc5696975)

[H. Current Health: Questions for Women Not Currently Pregnant, or are more than 12 months postpartum from most recent birth 38](#_Toc5696976)

[I. Lifetime health history: Questions for All Women 46](#_Toc5696978)

**NOTES:**

1. Women who are within 12 months of most recent birth AND report currently pregnant will complete section F (current pregnancy) not section H (current health <12 months postpartum)
2. Women who report child no longer in care 6 weeks after delivery will complete birth-related questions (skip feeding and colic questions), and if first live birth will complete First Live Birth pregnancy questions but NOT postpartum questions.

**Survey Introduction**

**Main Landing Page**

*Thank you for your interest in the Lifecourse Experiences and Pregnancy (LEAP) Study!*

*You have been invited to participate in this study because of your past involvement with the Project EAT study.*

*Information gathered from the LEAP survey will help us to better understand the ways in which different experiences over a woman’s life influence her health.*

*Please click the ‘Next Page’ button below to review the consent form and begin the survey.*

*If you have any questions, please contact us at* [*leap@umn.edu*](mailto:leap@umn.edu)*, 612-625-9833 or toll free 800-497-8636.*

**Survey Instructions & Consent Page**

*The LEAP Survey will take up to 45 minutes to complete. You will receive a $50 Amazon or Target gift card in the mail within 2 weeks after submitting your survey.*

*When taking the survey:*

- *Your answers will be saved each time you click “Next Page” or “Save & Return Later” at the bottom of a page.*
- *You may leave the survey at any time, and re-enter using the survey link provided to you in the email or letter we sent to you.*
- *If at any time, you choose not to participate in the study, close the browser window to exit.*
- *If you take this survey on a smart phone, you may find it helpful to turn your phone sideways to fully view the questions.*

*If you have any questions or technical problems with the survey, please contact us (*[*leap@umn.edu*](mailto:leap@umn.edu)*, 612-625-9833 or toll free at 800-497-8636).*

Please read the consent form attached here [PDF], and then check the box below if you choose to participate in the survey.

*□ I agree to participate in this research.*

[After checking the consent box and clicking “Next Page,” participant advances to the next screen – Gift Card & Contact Information.]

*“We want to be sure to send your gift card to the correct address. Please take a moment to indicate your gift card choice and verify the contact information we have on file for you. Please update or provide if missing.”*

[Available participant contact information (from the Access database) will be prepopulated in the REDCap survey page.]

Which type of gift card would you like to receive? [Radio button: $50 Amazon, $50 Target] **required*

Your first name [Prepopulated, Textbox] **required*

Your last name [Prepopulated, Textbox] **required*

Your street address (include Apt or Unit #) [Prepopulated, Textbox] **required*

City [Prepopulated, Textbox] **required*

State [Prepopulated, Drop down] **required*

Zipcode [Prepopulated, textbox] **required*

Email address [Prepopulated, Textbox]

Your preferred phone number [Prepopulated, Textbox]

Your alternate phone number [Prepopulated, Textbox]

# **A.** **Screening for Pregnancy and Births**

Note: All questions in this section are required (for participant to answer) in REDCap to allow correct branching/display of questions according to participant’s situation/responses.

1. Have you ever been pregnant? [Yes/No/I don’t know]

If YES, move on to next question.

If NO or I DON’T KNOW, move to F. Fertility section.

1. Are you currently pregnant? [Yes/No/I don’t know]
2. How many total pregnancies have you had, including pregnancies resulting in live births, stillbirths, miscarriages, or termination? (Do not count a current pregnancy) [Drop down, 0-15]
3. How many of your pregnancies have resulted in a live birth? If a multiple pregnancy (resulting in two or more babies), count as ONE birth. [Drop down, 0-15]

Note: If live birth response more than 6, will display *“For this survey, we’re interested in your first 6 live births*”.

Also, REDCap is programmed to check if # live births reported is > # pregnancies reported (Q3). If more live births reported than pregnancies, will display:

*“Please check your response. You indicated more live births than pregnancies. Please count a multiple pregnancy as ONE birth.”*

# **B. Pregnancy and postpartum health, about EACH LIVE BIRTH**

*The following questions ask about your live birth(s).*

1. When was your child(ren) from your [Xth live birth] born? [MM//DD/YYYY] [*Display calendar for date selection, program error check if 2019 entered]*
2. Was your [Xth] live birth a multiple birth (giving birth to or delivering two or more babies)? [Yes/No] ***Required in REDCap**

IF YES, what type of multiple [twins, triplets, quads, etc.]

1. IF NO to Q7 (SINGLETON):
   1. What is the name of your child from your [Xth] live birth? Please write ‘baby [X]’ if you prefer not to provide child’s name or child was not named. [Textbox]
   2. Approximately how much did [NAME, Xth live birth] weigh at birth? (For example: 7 pounds 8 ounces). *For reference,* *the average birth weight for babies is around 7.5 pounds, although between 5.5 and 10 pounds is considered normal. There are 16 ounces in 1 pound.*

i. Pounds [0-15, I don’t know]

ii. Ounces [0-15]

*If* ***I don’t know*** *[pounds] is selected, the following question will display below ounces:*

We know this information can be hard to remember.

At birth, would you say your baby was … [Radio buttons: very small, somewhat small, average size, somewhat large, very large]

- 1. What was [NAME, Xth live birth]’s sex at birth? [checkboxes: male/female/I don’t know/I prefer not to answer]
  2. Did [NAME] have any medical or health problems at birth? Check all that apply. [none, fever/infection, respiratory issues, heart problems, blood disorder, prematurity, required NICU or special care, other [textbox]]
  3. Was [NAME, Xth live birth] still living and in your care 6 weeks after their birth? [yes/no] ****Required* *in REDCap***
     - If yes, move on to 7f.
     - If no, skip 7f and 7g and move on to Q8.

*If only one live birth, go to section C (will ask about FLB pregnancy but will skip Section D -postpartum questions)

- 1. What type of milk did you feed [NAME] during his or her first four months? [Check all that apply: Breastmilk only, formula only, mostly breastmilk, mostly formula, half formula/half breastmilk, prefer not to answer]
  2. In the first 6 weeks of [NAME, Xth live birth]’s life, how true were the following statements? [Never, rarely, sometimes, usually, always true]
     1. He/she was happy most of the time
     2. He/she did not cry easily

IF YES to Q7 (MULTIPLES):

*Will ask 7a-g for each live birth (number based on response to type of multiple/number of babies].*

1. What is the name of your [first/second/third/fourth] multiple from your [Xth] live birth? Please write ‘baby [X]A,” “baby [X]B,” and so forth if you prefer not to provide child’s name or child was not named. [Textbox]
2. Approximately how much did [NAME Xth multiple of Xth live birth] weigh at birth? (For example: 7 pounds, 8 ounces). *For reference, the average birth weight for babies is around 7.5 pounds, although between 5.5 and 10 pounds is considered normal. There are 16 ounces in 1 pound.*

i. Pounds [0-15, I don’t know]

ii. Ounces [0-15]

*If* ***I don’t know*** *[pounds] is selected, the following question will display below ounces:*

We know this information can be hard to remember.

At birth, would you say your baby was … [Radio buttons: very small, somewhat small, average size, somewhat large, very large]

1. What was [NAME, Xth multiple of Xth live birth]’s sex at birth? [checkboxes: male/female/I don’t know/I prefer not to answer]
2. Did [NAME] have any medical or health problems at birth? [Check all that apply. [none, fever/infection, respiratory issues, heart problems, blood disorder, prematurity, required NICU or special care, other [textbox]]
3. Was [NAME, Xth multiple from Xth live birth] still living and in your care 6 weeks after their birth? [yes/no] ****Required in REDCap***
   - If yes, move on to 7f.
   - If no, skip 7f and 7g, go to next multiple from same birth
4. What type of milk did you feed [NAME, Xth multiple from Xth live birth] during his or her first four months? [Check all that apply: Breastmilk only, formula only, mostly breastmilk, mostly formula, half formula/half breastmilk, prefer not to answer]
5. In the first 6 weeks of [NAME, Xth multiple of Xth live birth]’s life, how true were the following statements? [Never, rarely, sometimes, usually, always true]
6. He/she was happy most of the time
7. He/she did not cry easily

*The next questions are about your weight before and during your pregnancy with [name or names]. We know that this information may be hard to recall. Please estimate as best you can. If you are not able to answer, please check 'I don't know'.*

1. About how much did you weigh, in pounds, just before this pregnancy? [Textbox w/#range 50-999 allowed; Checkbox “I don’t know”]

If ‘I don’t know’ checked, “*We understand this can be hard! If you can, we encourage you to make your best guess within the nearest 10 pounds.*”

1. About how much weight, in pounds, did you gain during this pregnancy? [Textbox w/#range 50-999 allowed; Checkbox “I don’t know”]

If ‘I don’t know’ checked, “*We understand this can be hard! If you can, we encourage you to to make your best guess within the nearest 10 pounds.*”

1. How many weeks pregnant were you when this baby was/these babies were born (i.e. gestational age at birth)? (For reference, 40 weeks is an average gestation for a singleton, and 38 weeks for twins.) [Textbox, limits to 20-45 weeks]
2. During this pregnancy were you ever told by a doctor, midwife, or other medical provider that you had any of the following conditions? Check all that apply. [Check boxes]
   1. Gestational diabetes (diabetes that started during pregnancy and ended after delivery)
   2. Pregnancy-related high blood pressure
   3. Pre-eclampsia/toxemia
   4. Hyperemesis gravidarum (excessive throwing up)
   5. Posttraumatic stress disorder, eating disorder or other psychiatric disorder (e.g., mood, anxiety)
   6. A condition that required bed rest
   7. Other medical conditions we should be aware of

*--> if other, please describe*

- 1. None

1. Did you have a cesarean section (c-section) with this birth? [Yes/No]

**SKIP Q13-16** **if < 12 months since this birth** [calculated based on Q5 birthdate]

1. In the 12 months after this birth, what were your main intentions with regard to your weight?
   1. I wanted to return to my pre-pregnancy weight
   2. I wanted to weigh more than my pre-pregnancy weight
   3. I wanted to weigh less than my pre-pregnancy weight
   4. I didn’t care about my weight
   5. Other [--> if other, please describe]
2. In the 12 months after this birth, what was your experience with weight?
   1. I was able to reach my desired weight without significant effort
   2. I was able to reach my desired weight with significant effort
   3. I wasn’t able to lose as much as intended
   4. I lost more weight than intended
   5. I didn’t focus on my weight
   6. Other [--> if other, please describe]
3. About how much did you weigh, in pounds, 12 months after this birth? [Textbox w/#range 50-999 allowed; Checkbox “I don’t know”]

If ‘I Don’t know’ checked, “We understand this can be hard! If you can, we encourage you to to make your best guess within the nearest 10 pounds.”

1. In the 12 months after this pregnancy were you told by a doctor, midwife, or other medical provider that you had any of the following conditions? Check all that apply. [Checkboxes]
2. Postpartum depression
3. Postpartum anxiety
4. Postpartum thyroid problems
5. Other medical conditions we should be aware of

--> if other, please describe

1. None

*“The following questions ask about prenatal and delivery care for your pregnancy with [name or names]. This information will be used if/when you choose to participate in the second phase of this study, which focuses on medical records.”*

1. Was this child born (or this birth) in Minnesota?

🡪 If YES:

- 1. What city did this birth occur? [textbox, write-in]
  2. Where did you receive prenatal care for this pregnancy? [Dropdown of common MN facilities (alpha order), I don’t know or remember, Private Practice, and Other; If Other or Private Practice – please describe]
  3. In what hospital or other birth facility did this birth occur? [Dropdown of common MN facilities (alpha order), I don’t know or remember, and Other; If Other – please describe]

🡪 If NO:

1. What state did this birth occur [dropdown of states, other, I don’t know or remember; if other, please describe]
2. What city did this birth occur? [textbox, write-in]
3. Where did you receive prenatal care for this pregnancy? [textbox write in, I don’t know or remember]
4. In what hospital or other birth facility did this birth occur? [textbox write in, I don’t know or remember]

***REPEAT SECTION B questions for EACH LIVE BIRTH before moving to Section C.**

**Thanks for your responses so far. You’re a third of the way done!**

## **C. Pregnancy psychosocial and behavioral factors, about FIRST LIVE BIRTH**

*We are interested in learning more about your pregnancy with [name_b1 or names of multiples]. The next set of questions will not be asked about any other births you may have had. (If you are currently pregnant, similar questions will be asked about your current pregnancy.)*

*We would like to know more about your weight status before you were pregnant.* *We know this information may be hard to recall. Please estimate as best you can. If you are not able to answer, please check 'I don't know'.*

1. In the five years prior to your pregnancy with your first live birth:
   1. How much did your weight fluctuate in pounds? [Drop down: 0-4 pounds, 5-9 pounds, 10-14 pounds, 15-19 pounds, 20 or more pounds, I don’t know]
   2. Approximately how much did you weigh at your heaviest in pounds when you were not pregnant? [textbox w/#range 50-999 allowed; Checkbox “I don’t know”]
   3. Approximately how much did you weigh at your lightest in pounds when you were not pregnant? [textbox w/#range 50-999 allowed; Checkbox “I don’t know”]
2. During your pregnancy with your first live birth, did your medical provider (doctor, midwife) provide any guidance on how much weight to gain during pregnancy? [Yes/No]

🡪 If Yes: What guidance did your provider give you? [Check boxes]

I was told how much weight I should gain; I was encouraged to gain more weight; I was encouraged to limit my weight gain; I was encouraged to not focus on my weight; Other [--> if other, please describe]

1. Aside from your medical provider, did anyone else give you information or advice on how much weight to gain? [Yes/No]

🡪If YES: a. Whose advice did you trust the most?

b. What did they tell you?

1. During your pregnancy with your first live birth, did you ever have trouble gaining weight or keeping weight on?

IF YES, which trimester? [check all that apply – 1,2,3]

1. During this pregnancy, did you ever try to limit the amount of weight you gained? [Yes/No]

IF YES, which trimester? [check all that apply – 1,2,3]

1. During this pregnancy, did you do any of the following in order to gain weight or keep weight on? Check all that apply. [Checkboxes]
   - Exercised less
   - Ate more high-fat foods
   - Ate more sweets
   - Drank soda pop and/or other sugar-sweetened beverages such as juice
   - Ate more snacks
   - Ate more frequently
   - Ate larger portions
   - Drank nutritional supplements such as Ensure, Boost or Carnation Instant Breakfast
   - Other [--> if other, please describe]]
   - None
2. During this pregnancy, did you do any of the following in order to keep from gaining too much weight? Check all that apply. [Checkboxes]
   - Exercised
   - Ate more fruits and vegetables
   - Limited high-fat foods
   - Limited sweets
   - Limited soda pop and other sugar-sweetened beverages
   - Watched my portion sizes (serving sizes)
   - Limited snacks
   - Limited eating out
   - Fasted
   - Ate very little food
   - Took diet pills
   - Made myself vomit (throw up)
   - Used diuretics
   - Used laxatives
   - Used food substitute (powder/special drink)
   - Skipped meals
   - Used tobacco products
   - Other [--> if other, please describe]
   - None
3. During your pregnancy with your first live birth, did you ever eat so much food in a short period of time that you would have been embarrassed if others saw you (binge eating)? [Yes/No]

🡪If YES: During the times that you ate this way, did you feel you couldn’t stop eating or control what or how much you were eating? [Yes/No]

1. How true were the following statements for you during your pregnancy with your first live birth? [Radio buttons: Never, seldom, sometimes, often, always]
   1. I spent a lot of time worrying about my pregnancy weight, shape, or size
   2. I felt unattractive because of my pregnancy weight, shape and size
   3. I was more concerned with how my pregnant body functioned than how it looked
   4. I felt happy with how my body functioned during pregnancy
   5. I felt happy with my pregnancy weight, shape, and size

*The next few questions ask about your physical activity levels before and during your pregnancy with your first live birth. Moderate physical activity refers to activities such as brisk walking and casual or easy bicycling. Vigorous physical activity refers to activities that produce large increases in breathing or heart rate, such as running; fast, long-distance or mountain bicycling; aerobic dance; or hiking uphill.*

1. In the 6 months before your pregnancy with your first live birth, how often did you participate in any moderate-vigorous physical activity for 20 minutes or more?

 Less than 1 day per week

 1 to 2 days per week

 3 to 4 days per week

 5 or more days per week

 I was told by a doctor, nurse, or other health care worker not to exercise

1. During this pregnancy, how often did you participate in any moderate-vigorous physical activity for 20 minutes or more?

 Less than 1 day per week

 1 to 2 days per week

 3 to 4 days per week

 5 or more days per week

 I was told by a doctor, nurse, or other health care worker not to exercise

1. Which of the following best describes your work status during the majority of your pregnancy with your first live birth? [Drop down]
2. Working for pay less than 30 hours per week
3. Working for pay 30-50 hours per week
4. Working for pay more than 50 hours per week
5. Not working for pay by choice (e.g., stay-at-home parent)
6. Unemployed
7. Were you a student during this pregnancy? [No, not a student/Yes, part-time/Yes, full-time]
8. During this pregnancy, did you receive any of the following types of public assistance? Check all that apply. [Check boxes]
9. Food assistance: SNAP (Supplemental Nutrition Assistance Program), WIC (Women, Infants, and Children), food from a food shelf/pantry
10. Temporary Assistance for Needy Families (TANF) also known as Welfare, General Assistance (GA), Supplemental Security Income (SSI)
11. Unemployment Benefits
12. Social Security Disability Benefits, Workers’ Compensation, Veterans Disability Benefits, or Disability Pension
13. Medicaid
14. Other [--> if other, please describe]
15. None
16. During this pregnancy, how hard was it for you to get by financially? [Drop down]
17. Not difficult at all
18. Somewhat difficult
19. Very difficult or can barely get by
20. Extremely difficult or impossible
21. Please indicate how often each statement was true for your household during your pregnancy with your first live birth: [Radio buttons with frequency: Often true, somewhat true, never true]
22. The food we bought just didn’t last, and we didn’t have money to get more
23. We couldn’t afford to eat balanced meals
24. During this pregnancy…
25. Did you or other adults in your household ever cut the size of your meals or skip meals because there wasn’t enough money for food? [Yes/No]
26. Did you ever eat less than you felt you should because there wasn’t enough money for food? [Yes/No]
27. Were you ever hungry but didn’t eat because there wasn’t enough money for food? [Yes/No]
28. What was your feeling about your pregnancy with your first live birth? [Drop down]
29. I intended to become pregnant when I did
30. I did not intend to become pregnant, but I was glad to become pregnant
31. I wanted to get pregnant someday but not when I did
32. I did not want to get pregnant then or at any time

🡪If a: Did you have any difficulty becoming pregnant? [Yes/No]

🡪If YES: Did you or your partner use any medical intervention (e.g., fertility drugs, in vitro fertilization) to help you become pregnant? [Yes/No]

1. Which of the following best describes your relationship status during your pregnancy with your first live birth? [Drop down]
2. Married, living with spouse
3. Unmarried, living with romantic partner
4. In a romantic relationship but not living together
5. Not in a romantic relationship
6. Other [--> if other, please describe]

- If YES to a-c, continue to 37
- If YES to d-e, skip to 38

*We would like to know more about your feelings while in this relationship. Your responses will be kept confidential.*

1. During your pregnancy with your first live birth … [Radio Buttons: Strongly disagree … Strongly agree]
2. My partner made me feel unsafe even in my own home
3. I felt ashamed of things my partner did to me
4. I tried not to rock the boat because I was afraid of what my partner might do
5. I felt owned and controlled by my partner
6. My partner could scare me without laying a hand on me

*We would also like to know about events you may have experienced during your pregnancy with your first live birth. Your responses will be kept confidential.*

1. During your pregnancy with your first live birth, did any of the following life events or problems happen to you? Check all that apply. [Check boxes]
2. You or your partner lost a job
3. You were divorced or separated from your partner
4. You experienced excessive credit card or student loan debt
5. You had to care for an ill family member
6. You were hit, slapped, kicked, or physically hurt by your spouse or partner
7. You were forced into sexual activities by your spouse or partner
8. You felt controlled, intimidated or threatened by your spouse or partner
9. You experienced inappropriate or unwanted sexual advances (not involving a spouse or partner)
10. You had a close family member or friend die violently, for example, in a serious car crash, mugging, homicide, suicide, or attack
11. You experienced another traumatic event in which you feared for your life or the life of a loved one (e.g., natural disaster, violent crime)
12. None of these

- If checked any, were any of the experiences so frightening, horrible, or upsetting that, during this pregnancy, you…[Yes/No]

i. Had nightmares about it or thought about it when you did not want to?

ii. Tried hard not to think about it or went out of your way to avoid situations that reminded you of it?

iii. Were constantly on guard, watchful, or easily startled?

iv. Felt numb or detached from others, activities, or your surroundings?

1. During your pregnancy with your first live birth, how often were you bothered or troubled by…[Radio buttons: Not at all… Very much]
2. Feeling too tired to do things
3. Having trouble going to sleep or staying asleep
4. Feeling unhappy, sad, or depressed
5. Feeling hopeless about the future
6. Feeling nervous or tense
7. Worrying too much about things
8. During this pregnancy … [Radio buttons: Never, once in a while, sometimes, a lot, most of the time, almost all the time]
   1. How frequently did you experience unfair treatment because you were a woman?
   2. How frequently did you witness unfair treatment toward another woman because of her gender?
   3. How frequently did you experience unfair treatment because you were pregnant?
   4. How frequently did you witness unfair treatment toward someone else because they were pregnant?
   5. Did you experience unfair treatment for any other reasons? [Yes/No]

🡪If response is greater than ‘Never’ or ‘Yes’:

For what reason(s)? [Check boxes] race, immigration status, language, gender identity, sexual orientation, weight, social class, other [--> if other, please describe]

*Some women may use tobacco, alcohol, prescription drugs and/or other types of drugs during pregnancy. The next set of questions will ask about your use of these products during your pregnancy with your first live birth. Your responses will be kept confidential and names will not be associated with any responses.*

1. During your pregnancy with your first live birth, did you ever use tobacco or nicotine-containing products (including e-cigarettes or vaping)? [Yes/No]

🡪If YES: What type of tobacco or nicotine-containing products? Check all that apply. [Checkboxes] cigarettes, e-cigarettes, vaping, other

1. During this pregnancy, did you ever drink alcohol? [Yes/No]

🡪 If NO, skip to 44.

🡪 If YES: How often on average did you drink alcohol during this pregnancy? By a drink we mean half an ounce of absolute alcohol (e.g. a 12 ounce can or glass of beer or cooler, a 5 ounce glass of wine, or a drink containing 1 shot of liquor). [Drop down] and continue to 43.

1. Every day
2. Nearly everyday
3. 3 or 4 days a week
4. 1 or 2 days a week
5. 3 or 4 days a month
6. About once a month
7. Less than once a month
8. During this pregnancy, what was the largest number of alcoholic drinks you had during a 24-hour period when you drank alcohol?
9. 12 or more drinks
10. 9 to 11 drinks
11. 7 to 8 drinks
12. 5 to 6 drinks
13. 3 to 4 drinks
14. 2 drinks
15. 1 drink
16. During your pregnancy with your first live birth, did you take any of the following medications regularly (at least once a week) for any reason? Check all that apply. [Checkboxes]
    1. Opioid pain medications such as hydrocodone (Vicodin), oxycodone (Percocet), codeine, or Dilaudid
    2. SSRIs (Selective Serotonin Reuptake Inhibitors) such as Sarafem, Zoloft, or Lexapro
    3. Other anti-depressants → *If other, please describe: [Textbox]*
    4. Benzodiazepines such as Klonopin, Xanax, or Valium
    5. Other anti-anxiety medications *→ If other, please describe: [Textbox]*
    6. Steroids such as prednisone
    7. Thyroid medications
    8. Anti-nausea/anti-emetic such as Zofran
    9. Other *→ If other, please describe: [Textbox]*
    10. None

- If YES to any, did you ever use [medication] in a way a doctor did not direct you to use it (e.g., used someone else’s prescription; took more than prescribed; took more frequently than prescribed)? [Yes/No]

1. During this pregnancy, did you ever take any of the following? Check all that apply.[Checkboxes]
   - Marijuana
   - Heroin/Fentanyl
   - Methamphetamine/psychostimulants
   - Prefer not to answer
   - None
2. During the trimester of this pregnancy when you had the most “morning sickness” (pregnancy-related nausea and vomiting), how many times a day on average did you vomit? [drop down, 0-10+]
3. For how many months of this pregnancy did you vomit at least once a day? [Drop down]
   1. None
   2. Less than 1 month
   3. 1-3 months
   4. 4-6 months
   5. Entire pregnancy

*Many women experience chronic pain at some point in their lives. By chronic pain we mean pain that lasted more than 3 months (e.g., back pain, migraines, pelvic pain, arthritis).*

1. During this pregnancy, did chronic pain interfere with your mood, activities, relationships, or sleep? [Yes/No]

If NO -> skip to 49

If YES -> continue to 48a

(a) At what age did you first start experiencing this pain?

1. In the 12 months prior to your pregnancy with your first live birth, how was your sleep quality overall? [Very good, Fairly good, Fairly bad, Very bad]
2. During this pregnancy, how was your sleep quality overall? [Very good, Fairly good, Fairly bad, Very bad]

# **D. Postpartum characteristics, about FIRST LIVE BIRTH**

1. Has it been 12 months or more since your first live birth? [Yes/No] ****Required in REDCap***

If NO 🡪 Will display section G questions at this point in REDCap and skip D questions

If YES 🡪 CONTINUE with section D questions

*The next set of questions are about the first 12 months after your pregnancy with [name or names if multiples]. We’re interesting in learning how this pregnancy influenced your health and well-being in the 12 months after this birth. Questions may be similar to those asked during your pregnancy.*

*In the 12 months after your first live birth …*

1. Relative to other parents with infants, how was your sleep quality overall?" [Very good, Fairly good, Fairly bad, Very bad]
2. In the 12 months after your first live birth, did you do any of the following in order to lose weight or keep from gaining weight? Check all that apply. [Check boxes]
   - Exercised
   - Ate more fruits and vegetables
   - Limited high-fat foods
   - Limited sweets
   - Limited soda pop or other sugar-sweetened beverages
   - Watched my portion sizes (serving sizes)
   - Limited snacks
   - Limited eating out
   - Limited alcohol
   - Fasted
   - Ate very little food
   - Took diet pills
   - Made myself vomit (throw up)
   - Used diuretics
   - Used laxatives
   - Used food substitute (powder/special drink)
   - Skipped meals
   - Used tobacco products
   - Other [--> if other, please describe]
   - None
3. In the 12 months after your first live birth, did you ever eat so much food in a short period of time that you would have been embarrassed if others saw you (binge eating)? [Yes/No]

🡪If YES: During the times that you ate this way, did you feel you couldn’t stop eating or control what or how much you were eating? [Yes/No]

1. In the 12 months after your first live birth, how true were the following statements for you? [Radio buttons: Never, seldom, sometimes, often, always]
2. I spent a lot of time worrying about my postpartum weight, shape, or size
3. I felt unattractive because of my postpartum weight, shape and size
4. I was more concerned with how my postpartum body functioned than how it looked
5. I felt happy with how my postpartum body functioned
6. I felt happy with my postpartum weight, shape, or size

*The next question asks about your physical activity level during the 12 months after this birth. Moderate physical activity refers to activities such as brisk walking and casual or easy bicycling. Vigorous physical activity refers to activities such as running; fast, long-distance or mountain bicycling; aerobic dance; or hiking uphill.*

1. In the 12 months after this birth, how often did you participate in any moderate-vigorous physical activity for 20 minutes or more?

 Less than 1 day per week

 1 to 2 days per week

 3 to 4 days per week

 5 or more days per week

 Other

*Many women experience chronic pain at some point in their lives. By chronic pain we mean pain that lasted more than 3 months (e.g., back pain, migraines, pelvic pain, arthritis).*

1. In the 12 months after this birth, did chronic pain interfere with your mood, activities, relationships, or sleep? [Yes/No]

If NO -> skip to 58

If YES -> continue to 57(a)

(a) At what age did you start experiencing this pain?

1. In the 12 months after your first live birth, how often were you bothered or troubled by…

[Radio buttons: Not at all … very much]

1. Feeling too tired to do things
2. Having trouble going to sleep or staying asleep
3. Feeling unhappy, sad, or depressed
4. Feeling hopeless about the future
5. Feeling nervous or tense
6. Worrying too much about things

**E. Questions about Current Pregnancy** (women indicating currently pregnant and may or may not have had a previous birth)

Current Pregnancy Characteristics

*“The following questions ask you about your current pregnancy.”*

1. Are you expecting twins, triplets, or other multiples? [Yes/No/Don’t Know]

🡪If YES: What type of multiple? [drop down]: twins, triplets, quads, I don’t know, other [Textbox]]

1. Do you know the sex of your baby (babies)? [check boxes]: male/female/Don’t know; provide multiple boxes for multiples]
2. What is your due date (MM/DD/YYYY)? [Textbox/open ended]
3. Are you receiving prenatal care for this pregnancy? [Yes/No]

-> If YES,

Are you receiving prenatal care in Minnesota?

If YES – In what facility are you receiving prenatal care? [Dropdown with common facilities in MN (alpha order), Private Practice, Other --> if other or private practice, please indicate name of provider and practice]

If NO – In what state are your receiving prenatal care? [dropdown states, ‘other’ [textbox write in]

1. During your current pregnancy so far have you been told that you have any of the following conditions? Check all that apply. [Check boxes]
2. Gestational diabetes (diabetes that starts during pregnancy and ends after delivery)
3. Pregnancy-related high blood pressure
4. Pre-eclampsia/toxemia
5. Hyperemesis gravidarum (excessive throwing up)
6. Posttraumatic stress disorder, eating disorder or other psychiatric disorder (e.g., mood, anxiety disorder)
7. A condition that requires bed rest
8. Other medical condition we should be aware of [--> if other, please describe]
9. None
10. About how much did you weigh just before this pregnancy began, in pounds? [Textbox w/#range 50-999 allowed; Checkbox “I don’t know”]

If ‘I don’t know’ checked, “We encourage you to make your best guess within the nearest 10 pounds.”

1. How much do you currently weigh, in pounds? [Textbox w/# range 50-999 allowed, Checkbox “I don’t know”]

If ‘I don’t know’ checked, “We encourage you to make your best guess within the nearest 10 pounds.”

1. About how tall are you?
   - 1. Feet [drop down, 3-7 range]
     2. Inches (round to the nearest inch) [drop down, 0-11 range]

1. During your current pregnancy so far, has your medical provider (doctor, midwife) given you any guidance on how much weight to gain during pregnancy? [Yes/No]

🡪 If Yes: What guidance did your provider give you? [Check boxes]

I was told how much weight I should gain; I was encouraged to gain more weight; I was encouraged to limit my weight gain; I was encouraged to not focus on my weight; Other [--> if other, please describe]

1. Aside from your medical provider, has anyone else given you information or advice on how much weight to gain? [Yes/No]

🡪If YES: a. Whose advice did you trust the most?

b. What did they tell you?

1. During your current pregnancy so far, have you ever had trouble gaining weight or keeping weight on? [Yes/No]

🡪IF YES, during which trimester? [check all that apply – 1,2,3]

1. During your current pregnancy so far, have you ever tried to limit the amount of weight you gained? [Yes/No]

🡪IF YES, during which trimester? [check all that apply – 1,2,3]

1. During your current pregnancy so far, have you done any of the following in order to gain weight or keep weight on? Check all that apply. [Check boxes]

- Exercise less
- Eat more high-fat foods
- Eat more sweets
- Drink soda pop or other sugar-sweetened beverages such as juice
- Eat larger portions
- Eat more snacks
- Eat more frequently
- Drink nutritional supplements such as Ensure, Boost or Carnation Instant Breakfast
- Other [--> if other, please describe]
- None

1. During your current pregnancy so far, have you done any of the following in order to keep from gaining too much weight? Check all that apply. [Check boxes]

- Exercised
- Ate more fruits and vegetables
- Limited high-fat foods
- Limited sweets
- Limited soda pop or other sugar-sweetened beverages
- Watched my portion sizes (serving sizes)
- Limited snacks
- Limited eating out
- Fasted
- Ate very little food
- Took diet pills
- Made myself vomit (throw up)
- Used diuretics
- Used laxatives
- Used food substitute (powder/special drink)
- Skipped meals
- Used tobacco products
- Other [--> if other, please describe]
- None

1. During your current pregnancy so far, have you ever eaten so much food in a short period of time that you would have been embarrassed if others saw you (binge eating)? [Yes/No]

🡪If YES: During the times that you ate this way, did you feel you couldn’t stop eating or control what or how much you were eating? [Yes/No]

1. How true are the following statements for you during your current pregnancy so far? [Radio buttons: Never, seldom, sometimes, often, always]
2. I spend a lot of time worrying about my pregnancy weight, shape, or size
3. I feel unattractive because of my pregnancy weight, shape and size
4. I am more concerned with how my pregnant body functions than how it looks
5. I feel happy with how my body functions during pregnancy
6. I feel happy with my pregnancy weight, shape, and size

*The next few questions ask about your physical activity levels before and during your current pregnancy. Moderate physical activity refers to activities such as brisk walking and casual or easy bicycling. Vigorous physical activity refers to activities such as running; fast, long-distance or mountain bicycling; aerobic dance; or hiking uphill.*

1. In the 6 months before this pregnancy, how often did you participate in moderate-vigorous physical activity for 20 minutes or more?

 Less than 1 day per week

 1 to 2 days per week

 3 to 4 days per week

 5 or more days per week

 I was told by a doctor, nurse, or other health care worker not to exercise

1. During your current pregnancy so far, how often have you participated in moderate-vigorous physical activity for 20 minutes or more?

 Less than 1 day per week

 1 to 2 days per week

 3 to 4 days per week

 5 or more days per week

 I was told by a doctor, nurse, or other health care worker not to exercise

“*We would like to know a little more about your well-being during your current pregnancy.”*

1. Which of the following best describes your work status during the majority of this pregnancy? [Drop down]
2. Working for pay <30 hours per week
3. Working for pay 30-50 hours per week
4. Working for pay >50 hours per week
5. Not working for pay by choice (e.g., stay-at-home parent)
6. Unemployed
7. Have you been a student during this pregnancy? [No, not a student/Yes, part-time/Yes, full-time]
8. During your current pregnancy so far, have you received any of the following types of public assistance? Check all that apply. [Check boxes]
9. Food assistance: SNAP (Supplemental Nutrition Assistance Program), WIC (Women, Infants, and Children), food from a food shelf/pantry
10. Temporary Assistance for Needy Families (TANF) also known as Welfare, General Assistance (GA), Supplemental Security Income (SSI)
11. Unemployment Benefits
12. Social Security Disability Benefits, Workers’ Compensation, Veterans Disability Benefits, or Disability Pension
13. Medicaid
14. Other [--> if other, please describe]
15. None
16. During your current pregnancy so far, how hard has it been for you to get by financially? [Drop down]
17. Not difficult at all
18. Somewhat difficult
19. Very difficult or can barely get by
20. Extremely difficult or impossible
21. During your current pregnancy so far, how often have the following statements been true for your household? [Radio buttons with frequency: Often true, somewhat true, never true]
22. The food we bought just didn’t last, and we didn’t have money to get more
23. We couldn’t afford to eat balanced meals
24. During your current pregnancy so far …
25. Have you or other adults in your household ever cut the size of your meals or skipped meals because there wasn’t enough money for food? [Yes/No]
26. Have you ever eaten less than you felt you should because there wasn’t enough money for food? [Yes/No]
27. Have you ever been hungry but didn’t eat because there wasn’t enough money for food? [Yes/No]
28. What is your feeling about this pregnancy? [Drop down]
29. I intended to become pregnant when I did
30. I did not intend to become pregnant, but I was glad to become pregnant
31. I wanted to get pregnant someday but not when I did
32. I did not want to get pregnant at the moment or at any time

🡪If a: Did you have any difficulty becoming pregnant? [Yes/No]

🡪If YES: Did you or your partner use any medical intervention (e.g., fertility drugs, in vitro fertilization) to help you become pregnant? [Yes/No]

1. Which of the following best describes your relationship status during your pregnancy so far? [Drop down]
2. Married, living with spouse
3. Unmarried, living with romantic partner
4. In a romantic relationship but not living together
5. Not in a romantic relationship
6. Other [--> if other, please describe]

- If YES to a-c, continue to 85
- If YES to d-e, skip to 86

*We would like to know more about your feelings while in this relationship. Your responses will be kept confidential. A list of support resources can be found here* [attachment of Support Resources will open in a new window].

1. During your current pregnancy so far … [Radio Buttons: Strongly Disaree …Strongly Agree]
2. My partner has made me feel unsafe even in my own home
3. I have felt ashamed of things my partner did to me
4. I have tried not to rock the boat because I am afraid of what my partner might do
5. I have felt owned and controlled by my partner
6. My partner can scare me without laying a hand on me

**You’re more than halfway done, keep going to earn your $50 gift card!**

*We would also like to know about events you may have experienced during your current pregnancy. Your responses will be kept confidential.*

1. During your current pregnancy so far, have any of the following life events or problems happened to you? Check all that apply. [Check boxes]
2. You or your partner lost a job
3. You were divorced or separated from your partner
4. You experienced excessive credit card or student loan debt
5. You had to care for an ill family member
6. You were hit, slapped, kicked, or physically hurt by your spouse or partner
7. You were forced into sexual activities by your spouse or partner
8. You felt controlled, intimidated or threatened by your spouse or partner
9. You experienced inappropriate or unwanted sexual advances (not involving a spouse or partner)
10. You had a close family member or friend die violently, for example, in a serious car crash, mugging, homicide, suicide, or attack
11. You experienced another traumatic event in which you feared for your life or the life of a loved one (e.g., natural disaster, violent crime)
12. None of these

- If checked any, were any of the above experiences, or other traumatic experiences you have had in your life so frightening, horrible, or upsetting that, during this pregnancy, you…[Yes/No]

i. Had nightmares about it or thought about it when you did not want to?

ii. Tried hard not to think about it or went out of your way to avoid situations that reminded you of it?

iii. Were constantly on guard, watchful, or easily startled?

iv. Felt numb or detached from others, activities, or your surroundings?

*** If any are checked in Q86, display Support Resources** (as a new REDCap page)

The following services are available 24/7 to provide confidential support to individuals in distress, crisis or concerned relationships.

Minnesota Day One® Crisis Line (<https://dayoneservices.org/shelters-support/>)

1-866-223-1111; Text 612-399-9995

Greater Minneapolis Crisis Nursery (<http://crisisnursery.org>)

763-591-0100 (hotline)

Offers free, voluntary services for parents who face barriers such as unemployment, homelessness,

medical or mental health concerns, poverty, domestic violence, or other difficult situations.

Crisis Text Line (<https://www.crisistextline.org/>)

Text HOME to 741741 in the US

National Domestic Violence Hotline (<https://www.thehotline.org/>)

1-800-799-7233

National Suicide Prevention Lifeline (<https://suicidepreventionlifeline.org/>)

1-800-273-8255

National Drug Helpline (<http://drughelpline.org/>)

1-888-633-3239

Substance Abuse and Mental Health Services Administration’s National Helpline (<https://www.samhsa.gov/find-help/national-helpline>)

1-800-662-HELP (4357)

1. During your current pregnancy so far how often have you been bothered or troubled by…[Radio buttons: Not at all… Very much]
2. Feeling too tired to do things
3. Having trouble going to sleep or staying asleep
4. Feeling unhappy, sad, or depressed
5. Feeling hopeless about the future
6. Feeling nervous or tense
7. Worrying too much about things
8. During your current pregnancy so far … [Radio buttons: Never, once in a while, sometimes, a lot, most of the time, almost all the time]
   1. How frequently have you experienced unfair treatment because you are a woman?
   2. How frequently have you witnessed unfair treatment toward another woman because of her gender?
   3. How frequently have you experienced unfair treatment because you are pregnant?
   4. How frequently have you witnessed unfair treatment toward someone else because they are pregnant?
   5. Have you experienced unfair treatment for any other reasons? [Yes/No]

🡪If response greater than ‘Never’ or ‘Yes’:

For what reason(s)? [Checkboxes] Race, immigration status, language, gender identity, sexual orientation, weight, social class, other [--> if other, please describe]

*Some women may at times in their life use tobacco, alcohol, prescription drugs and/or other types of drugs. The next set of questions will ask about your use of these products during the few months before your current pregnancy. Your responses will be kept confidential and names will not be associated with any responses.*

1. In the 3 months before you knew you were pregnant, did you ever use tobacco or nicotine containing product (including e-cigarettes or vaping)? [Yes/No]

🡪If YES: What type of tobacco or nicotine-containing products? Check all that apply. [Checkboxes] cigarettes, e-cigarettes, vaping, other

1. In the 3 months before you knew you were pregnant, did you ever drink alcohol? [Yes/No]

🡪 If NO, skip to 92.

🡪 If YES: How often on average did you drink alcohol? By a drink we mean half an ounce of absolute alcohol (e.g. a 12 ounce can or glass of beer or cooler, a 5 ounce glass of wine, or a drink containing 1 shot of liquor)

[Drop down] and continue to 91.

1. Every day
2. Nearly everyday
3. 3 or 4 days a week
4. 1 or 2 days a week
5. 3 or 4 days a month
6. About once a month
7. Less than once a month
8. In the 3 months before you knew you were pregnant, what was the largest number of alcoholic drinks you had during a 24-hour period when you drank alcohol?
9. 12 or more drinks
10. 9 to 11 drinks
11. 7 to 8 drinks
12. 5 to 6 drinks
13. 3 to 4 drinks
14. 2 drinks
15. 1 drink
16. In the 3 months before you knew you were pregnant, did you take any of the following medications (at least once a week) for any reason? Check all that apply. [Yes/No]
17. Opioid pain medications such as hydrocodone (Vicodin), oxycodone (Percocet), codeine, or Dilaudid
18. SSRIs (Selective serotonin reuptake inhibitors) such as Sarafem, Zoloft, or Lexapro
19. Other anti-depressants → If other, please describe: [Textbox]
20. Benzodiazepines such as Klonopin, Xanax, or Valium
21. Other anti-anxiety medications → If other, please describe: [Textbox]
22. Steroids such as prednisone
23. Thyroid medications
24. Anti-nausea/anti-emetic such as Zofran
25. Other → If other, please describe: [Textbox]
26. None

- If YES to any, did you ever use [mediation] in a way a doctor did not direct you to use it (e.g., used someone else’s prescription; took more than prescribed; took more frequently than prescribed)? [Yes/No]

1. In the 3 months before you knew you were pregnant, did you take any of the following? Check all that apply. [Checkboxes]

- Marijuana
- Heroin/Fentanyl
- Methamphetamine/Psychostimulants
- Prefer not to answer
- None

1. During your current pregnancy so far when you had the most “morning sickness” (pregnancy-related nausea and vomiting), how many times a day on average did you vomit? [0-10+]
2. Over the past week, have you experienced any “morning sickness”? [Yes/No]

*Many women experience chronic pain at some point in their lives. By chronic pain we mean pain that lasted more than 3 months (e.g., back pain, migraines, pelvic pain, arthritis).*

1. During your current pregnancy so far, has chronic pain interfered with your mood, activities, relationships, or sleep? [Yes/No]

If NO -> skip to 97

If YES -> continue to 96a

(a) Select the best number that describes how, during your pregnancy so far, chronic pain interfered with your [0 Does not interfere to 5 Completely interferes]:

- 1. General activity
  2. Mood
  3. Walking ability
  4. Sleep
  5. Enjoyment of life

🡪 If YES or >0 to any -> continue to 96b

(b) At what age did you first experience this pain?

(c) Did you use any of the following to manage your pain? [Check-boxes]

Over-the-counter medications, prescription medication, physical therapy, meditation/yoga/other holistic therapy (acupuncture or massage), Other [--> if other, please describe]

“*We would like to know a little more about your sleep quality during your current pregnancy.”*

1. In the 12 months prior to this pregnancy, how was your sleep quality overall? [Very good, Fairly good, Fairly bad, Very bad]
2. During this pregnancy, how has your sleep quality been overall? [Very good, Fairly good, Fairly bad, Very bad]

# **F. Questions about infertility** (women indicating no births and not currently pregnant)

*The next set of questions ask about your experience with trying to get pregnant and fertility treatments, if applicable.*

1. Have you ever tried to get pregnant? [Yes/No]

- If NO🡪Skip section F and go to section H

1. Are you currently trying to get pregnant? [Yes/No]
2. What were the outcomes of your attempts to get pregnant?
   1. I have not yet gotten pregnant
   2. I got pregnant but miscarried
   3. Other [--> if other, please describe]
3. Does your insurance cover fertility-enhancing drugs or other fertility treatments? [Yes, fully covered; Yes, partially covered; No; I don’t know; Other [text box]]
4. Have you ever taken any fertility-enhancing drugs or received other fertility treatments? [Yes/No]

If NO, please describe why: [Check boxes] Insurance coverage, concern about procedure, money, my beliefs and/or partner/family, felt treatment was not safe, other [--> if other, please describe]

1. Did a medical provider ever tell you or your partner that you had any of the following? Check all that apply. [Check boxes]

- Endometriosis
- Polycystic ovaries
- Uterine fibroids
- No or abnormal ovulation
- Low levels of reproductive hormones (progesterone, estrogen)
- Scar tissue in the pelvis or fallopian tubes
- Male infertility
- Other health issues that may have prevented pregnancy [--> if other, please describe]
- None

1. Do you have any adopted or step-children? [Yes/No]

If YES -> Adopted children [0-10]

Step-children [0-10]

# **G. Current Health: Questions for Women within 12 months postpartum from most recent birth**

1. Has it been more than 12 months since your most recent birth? [Yes/No] ****Required in REDCap***

If NO 🡪 continue with Section G

If YES 🡪 Skip section G and go to Section H

*We’re interested in learning how your most recent birth has influenced your health and well-being in the past 12 months. Questions asked here may be similar to those asked previously about your pregnancy.*

1. Relative to other parents with infants, how has your sleep quality been overall since your most recent birth?" [Very good, Fairly good, Fairly bad, Very bad]

*The next questions are about your weight since your most recent birth. We know this information may be hard to recall. Please estimate as best you can. If you are not able to answer, please check 'I don't know'.*

1. What is your current weight in pounds? [textbox w/#range 50-999 allowed; Checkbox “I don’t know”]

If “I don’t know” selected, “We encourage you to make your best guess within the nearest 10 pounds” displays.

1. About how tall are you?
2. Feet [drop down, 3-7 range]
3. Inches (round to the nearest inch) [drop down, 0-11 range]
4. Since your most recent birth, have you done any of the following in order to lose weight or keep from gaining weight? Check all that apply. [Check boxes]

- Exercised
- Ate more fruits and vegetables
- Limited high-fat foods
- Limited sweets
- Limited soda pop or other sugar-sweetened beverages
- Watched my portion sizes (serving sizes)
- Limited snacks
- Limited eating out
- Limited alcohol
- Fasted
- Ate very little food
- Took diet pills
- Made myself vomit (throw up)
- Used diuretics
- Used laxatives
- Used food substitute (powder/special drink)
- Skipped meals
- Used tobacco products
- Other [--> if other, please describe]
- None

1. Since your most recent birth, have you ever eaten so much food in a short period of time that you would have been embarrassed if others saw you (binge eating)? [Yes/No]

🡪If YES: During the times that you ate this way, did you feel you couldn’t stop eating or control what or how much you were eating? [Yes/No]

1. Since your most recent birth, how true have the following statements been for you? [Radio buttons: Never … Always]
2. I have spent a lot of time worrying about my postpartum weight, shape, or size
3. I have felt unattractive because of my postpartum weight, shape and size
4. I have been more concerned with how my postpartum body functions than how it looks
5. I have felt happy with how my postpartum body functions
6. I have felt happy with my postpartum weight, shape, or size

*The next question asks about your physical activity level during the month(s) after this birth. Moderate physical activity refers to activities such as brisk walking and casual or easy bicycling. Vigorous physical activity refers to activities such as running; fast, long-distance or mountain bicycling; aerobic dance; or hiking uphill.*

1. Since your most recent birth, how often have you participated in any moderate-vigorous physical activity for 20 minutes or more?

 Less than 1 day per week

 1 to 2 days per week

 3 to 4 days per week

 5 or more days per week

 Other

*Many women experience chronic pain at some point in their lives. By chronic pain we mean pain that lasted more than 3 months (e.g., back pain, migraines, pelvic pain, arthritis).*

1. Since your most recent birth, has chronic pain interfered with your mood, activities, relationships, or sleep? [Yes/No]

If NO -> skip to 115

If YES -> continue to 114(a)

(a) Select the best number that describes how, since your most recent birth, chronic pain has interfered with your [0 Does not interfere to 5 Completely interferes]:

1. General activity
2. Mood
3. Walking ability
4. Sleep
5. Enjoyment of life

- If YES or >0 -> continue to 114b

(b) At what age did you first experience this pain?

(c) Have you used any of the following to manage your pain? [Check-boxes]

Over the-counter medications, prescription medication, physical therapy, meditation/yoga/other holistic therapy (acupuncture or massage), Other [--> if other, please describe]

*Psychosocial Well-being in the Postpartum Period*

1. Since your most recent birth, how often have you been bothered or troubled by… [Radio buttons: Not at all… Very much]
2. Feeling too tired to do things
3. Having trouble going to sleep or staying asleep
4. Feeling unhappy, sad, or depressed
5. Feeling hopeless about the future
6. Feeling nervous or tense
7. Worrying too much about things
8. Since your most recent birth …[Radio buttons: Never, once in a while, sometimes, a lot, most of the time, almost all the time]
   1. How frequently have you experienced unfair treatment because you are a woman?
   2. How frequently have you witnessed unfair treatment toward another woman because of her gender?
   3. How frequently have you experienced unfair treatment because you are a mother?
   4. How frequently have you witnessed unfair treatment toward someone else because they are a mother?
   5. Have you experienced unfair treatment for any other reasons? [Yes/No]

🡪If response greater than ‘Never’ or ‘Yes’:

For what reason(s)? [Check boxes] race, immigration status, language, gender identity, sexual orientation, weight, social class, other [Textbox]

1. Since your most recent birth, which of the following best describes your current work status? [Drop down]
2. Working for pay less than 30 hours per week
3. Working for pay 30-50 hours per week
4. Working for pay more than 50 hours per week
5. Not working for pay by choice (e.g., stay-at-home parent)
6. Unemployed
7. On maternity or parental leave from work
8. Since your most recent birth, have you been a student? [No, not a student/Yes, part-time/Yes, full-time]
9. Since your most recent birth, have you received any of the following types of public assistance? Check all that apply. [Check boxes]
10. Food assistance: SNAP (Supplemental Nutrition Assistance Program), WIC (Women, Infants, and Children), food from a food shelf/pantry
11. Temporary Assistance for Needy Families (TANF) also known as Welfare, General Assistance (GA), Supplemental Security Income (SSI)
12. Unemployment Benefits
13. Social Security Disability Benefits, Workers’ Compensation, Veterans Disability Benefits, or Disability Pension
14. Medicaid
15. Other [--> if other, please describe]
16. None
17. Since your most recent birth, how hard has it been for you to get by financially? [Drop down]
18. Not difficult at all
19. Somewhat difficult
20. Very difficult or can barely get by
21. Extremely difficult or impossible
22. Since your most recent birth, how often have the following statements been true for your household? [Radio buttons with frequency: Often true, somewhat true, never true]
23. The food we bought just didn’t last, and we didn’t have money to get more
24. We couldn’t afford to eat balanced meals
25. Since your most recent birth …
26. Have you or other adults in your household ever cut the size of your meals or skipped meals because there wasn’t enough money for food? [Yes/No]
27. Have you ever eaten less than you felt you should because there wasn’t enough money for food? [Yes/No]
28. Have you ever been hungry but didn’t eat because there wasn’t enough money for food? [Yes/No]
29. Which of the following best describes your current relationship status: [Drop down]

a. Married, living with spouse

b. Unmarried, living with romantic partner

c. In a romantic relationship but not living together

d. Not in a romantic relationship

e. Other [--> if other, please describe]

-> If YES to a-c, continue to 124

-> If YES to d-e, skip to 125

*We would like to know more about your feelings while in this relationship. Your responses will be kept confidential. A list of support resources can be found here* [attachment of Support Resources will open in a new window].

1. Since your most recent birth …[Radio Buttons: Strongly Disagree …Strongly Agree]
2. My partner has made me feel unsafe even in my own home
3. I have felt ashamed of things my partner did to me
4. I’ve tried not to rock the boat because I was afraid of what my partner might do
5. I’ve felt owned and controlled by my partner
6. My partner can scare me without laying a hand on me

*We would also like to know about events you may have experienced since your most recent birth. Your responses will be kept confidential.*

1. Since your most recent birth, have any of the following life events or problems happened to you? Check all that apply. [Check boxes]
2. You or your partner lost a job
3. You were divorced or separated from your partner
4. You experienced excessive credit card or student loan debt
5. You had to care for an ill family member
6. You were hit, slapped, kicked, or physically hurt by your spouse or partner
7. You were forced into sexual activities by your spouse or partner
8. You felt controlled, intimidated or threatened by your spouse or partner
9. You experienced inappropriate or unwanted sexual advances (not involving a spouse or partner)
10. You had a close family member or friend die violently, for example, in a serious car crash, mugging, homicide, suicide, or attack
11. You experienced another traumatic event in which you feared for your life or the life of a loved one (e.g., natural disaster, violent crime)
12. None of these

- If checked any, were any of the above experiences, or other traumatic experiences you have had in your life so frightening, horrible, or upsetting that, since this birth, you…[Yes/No]

i. Had nightmares about it or thought about it when you did not want to?

ii. Tried hard not to think about it or went out of your way to avoid situations that reminded you of it?

iii. Were constantly on guard, watchful, or easily startled?

iv. Felt numb or detached from others, activities, or your surroundings?

*** If any are checked in Q125, display Support Resources** (as a new REDCap page)

The following services are available 24/7 to provide confidential support to individuals in distress, crisis or concerned relationships.

Minnesota Day One® Crisis Line (<https://dayoneservices.org/shelters-support/>)

1-866-223-1111; Text 612-399-9995

Greater Minneapolis Crisis Nursery (<http://crisisnursery.org>)

763-591-0100 (hotline)

Offers free, voluntary services for parents who face barriers such as unemployment, homelessness,

medical or mental health concerns, poverty, domestic violence, or other difficult situations.

Crisis Text Line (<https://www.crisistextline.org/>)

Text HOME to 741741 in the US

National Domestic Violence Hotline (<https://www.thehotline.org/>)

1-800-799-7233

National Suicide Prevention Lifeline (<https://suicidepreventionlifeline.org/>)

1-800-273-8255

National Drug Helpline (<http://drughelpline.org/>)

1-888-633-3239

Substance Abuse and Mental Health Services Administration’s National Helpline (<https://www.samhsa.gov/find-help/national-helpline>)

1-800-662-HELP (4357)

*Some women may at times in their life use tobacco, alcohol, prescription drugs and/or other types of drugs. The next set of questions will ask about your use of these products since your most recent birth. Your responses will be kept confidential and names will not be associated with any responses.*

1. Since your most recent birth, have you used tobacco or nicotine-containing products (including e-cigarettes)? [Yes/No]

→If YES: What type of tobacco or nicotine-containing products? Check all that apply. [Checkboxes] cigarettes, e-cigarettes, vaping, other

1. Since your most recent birth, did you ever drink alcohol? [Yes/No]

🡪 If NO, skip to 129.

🡪 If YES: How often on average did you drink alcohol? By a drink we mean half an ounce of absolute alcohol (e.g. a 12 ounce can or glass of beer or cooler, a 5 ounce glass of wine, or a drink containing 1 shot of liquor).

[Drop down] and continue to 128.

1. Every day
2. Nearly everyday
3. 3 or 4 days a week
4. 1 or 2 days a week
5. 3 or 4 days a month
6. About once a month
7. Less than once a month
8. Since your most recent birth, what was the largest number of alcoholic drinks you had during a 24-hour period when you drank alcohol?
9. 12 or more drinks
10. 9 to 11 drinks
11. 7 to 8 drinks
12. 5 to 6 drinks
13. 3 to 4 drinks
14. 2 drinks
15. 1 drink
16. Since your most recent birth, have you taken any of the following medications (at least once a week) for any reason? Check all that apply. [Yes/No]
17. Opioid pain medications such as hydrocodone (Vicodin), oxycodone (Percocet), codeine, or Dilaudid
18. SSRIs (Selective serotonin reuptake inhibitors) such as Sarafem, Zoloft, or Lexapro
19. Other anti-depressants → *If other, please describe: [Textbox]*
20. Benzodiazepines such as Klonopin, Xanax, or Valium
21. Other anti-anxiety medications → *If other, please describe: [Textbox]*
22. Steroids such as prednisone
23. Thyroid medications
24. Anti-nausea/anti-emetic such as Zofran
25. Other → *If other, please describe: [Textbox]*
26. None

- If YES to any, did you ever use [medication] in a way a doctor did not direct you to use it (e.g., used someone else’s prescription; took more than prescribed; took more frequently than prescribed)? [Yes/No]

1. Since your most recent birth, have you taken any of the following? Check all that apply. [Checkboxes]

- Marijuana
- Heroin/Fentanyl
- Methamphetamine/Psychostimulants
- Prefer not to answer
- None

**You’re more than halfway done, keep going to earn your $50 gift card!**

# **H. Current Health: Questions for Women who are Not Currently Pregnant and are more than 12 months postpartum from most recent birth**

**NOTE: Women who are < 12 months postpartum from most recent birth will SKIP this section, and instead complete section G.**

For women who reported no live births (and thus didn’t complete First Live Birth section)

*We’re interested in learning about your health and well-being in the past 12 months.*

For women who completed the first live birth pregnancy/postpartum sections –

*We’re interested in learning about your health and well-being in the past 12 months.*

*Questions asked here may be similar to those previously asked about your pregnancy that resulted in your first live birth.*

*We would like to know about your weight in the past 12 months. We know this information may be hard to recall. Please estimate as best you can. If you are not able to answer, please check 'I don't know'.*

1. What is your current weight in pounds? [textbox w/# range 50-999 allowed; Checkbox “I don’t know”]

If select “I don’t know,” display “We encourage you to make your best guess within the nearest 10 pounds.”

1. How tall are you?
2. Feet [drop down, 3-7 range]
3. Inches (round to the nearest inch) [drop down, 0-11 range]

*The next set of questions are about your health behaviors in the past 12 months.*

1. In the past 12 months, did you do any of the following in order to lose weight or keep from gaining weight? Check all that apply. [Checkboxes]
   - Exercised
   - Ate more fruits and vegetables
   - Limited high-fat foods
   - Limited sweets
   - Limited soda pop or other sugar-sweetened beverages
   - Watched my portion sizes (serving sizes)
   - Limited snacks
   - Limited eating out
   - Limited alcohol
   - Fasted
   - Ate very little food
   - Took diet pills
   - Made myself vomit (throw up)
   - Used diuretics
   - Used laxatives
   - Used food substitute (powder/special drink)
   - Skipped meals
   - Used tobacco products
   - Other [--> if other, please describe]
   - None
2. In the past 12 months, did you ever eat so much food in a short period of time that you would have been embarrassed if others saw you (binge eating)? [Yes/No]

🡪If YES: During the times that you ate this way, did you feel you couldn’t stop eating or control what or how much you were eating? [Yes/No]

1. In the past 12 months, how true were the following statements? [Radio buttons: Never, seldom, sometimes, often, always]
2. I spent a lot of time worrying about my weight, shape, or size
3. I felt unattractive because of my weight, shape and size
4. I was more concerned with how my body functioned than how it looked
5. I felt happy with how my body functioned
6. I felt happy with my weight, shape, and size
7. In the past 12 months, how satisfied have you been with your: [1= very dissatisfied to 5= very satisfied]
   1. Weight
   2. Body shape
   3. Waist
   4. Hips
   5. Thighs
   6. Stomach

*The next question asks about your physical activity level over the last 12 months. Moderate physical activity refers to activities such as brisk walking and casual or easy bicycling. Vigorous physical activity refers to activities such as running; fast, long-distance or mountain bicycling; aerobic dance; or hiking uphill.*

1. In the past 12 months, how often did you participate in any moderate-vigorous physical activity for 20 minutes or more?

 Less than 1 day per week

 1 to 2 days per week

 3 to 4 days per week

 5 or more days per week

 I was told by a doctor, nurse, or other health care worker not to exercise

*The next set of questions ask about your health and well-being in the past 12 months.*

1. Which of the following best describes your current work status? [Drop down]
2. Working for pay <30 hours per week
3. Working for pay 30-50 hours per week
4. Working for pay >50 hours per week
5. Not working for pay by choice (e.g., stay-at-home parent)
6. Unemployed
7. Have you been a student in the past 12 months? [No, not a student/Yes, part-time/Yes, full-time]
8. In the past 12 months, have you received any of the following types of public assistance? [Check boxes]
9. Food assistance: SNAP (Supplemental Nutrition Assistance Program), WIC (Women, Infants, and Children), food from a food shelf/pantry
10. Temporary Assistance for Needy Families (TANF) also known as Welfare, General Assistance (GA), Supplemental Security Income (SSI)
11. Unemployment Benefits
12. Social Security Disability Benefits, Workers’ Compensation, Veterans Disability Benefits, or Disability Pension
13. Medicaid
14. Other [--> if other, please describe]
15. None
16. In the past 12 months, how hard has it been for you to get by financially? [Drop down]
17. Not difficult at all
18. Somewhat difficult
19. Very difficult or can barely get by
20. Extremely difficult or impossible
21. In the past 12 months, how often have the following statements true for your household? [Radio buttons with frequency: Often true, somewhat true, never true]
22. The food we bought just didn’t last, and we didn’t have money to get more
23. We couldn’t afford to eat balanced meals
24. In the past 12 months…
25. Have you or other adults in your household ever cut the size of your meals or skipped meals because there wasn’t enough money for food? [Yes/No]
26. Have you ever eaten less than you felt you should because there wasn’t enough money for food? [Yes/No]
27. Have you ever been hungry but didn’t eat because there wasn’t enough money for food? [Yes/No]
28. Which of the following best describes your current relationship status: [Drop down]

a. Married, living with spouse

b. Unmarried, living with romantic partner

c. In a romantic relationship but not living together

d. Not in a romantic relationship

e. Other [--> if other, please describe]

-> If YES to a-c, continue to 145

-> If YES to d-e, skip to 146

*We would like to know more about your feelings while in this relationship. Your responses will be kept confidential. A list of support resources can be found here* [attachment of Support Resources will open in a new window].

1. In the past 12 months…[Radio Buttons: Strongly Disagree … Strongly Agree]
2. My partner has made me feel unsafe even in my own home
3. I have felt ashamed of things my partner did to me
4. I’ve tried not to rock the boat because I was afraid of what my partner might do
5. I’ve felt owned and controlled by my partner
6. My partner can scare me without laying a hand on me

*We would also like to know about events you may have experienced in the past 12 months. Your responses will be kept confidential.*

1. In the past 12 months, have any of the following life events or problems happened to you? Check all that apply. [Check boxes]
2. You or your partner lost a job
3. You were divorced or separated from your partner
4. You experienced excessive credit card or student loan debt
5. You had to care for an ill family member
6. You were hit, slapped, kicked, or physically hurt by your spouse or partner
7. You were forced into sexual activities by your spouse or partner
8. You felt controlled, intimidated or threatened by your spouse or partner
9. You experienced inappropriate or unwanted sexual advances (not involving a spouse or partner)
10. You had a close family member or friend die violently, for example, in a serious car crash, mugging, homicide, suicide, or attack
11. You experienced another traumatic event in which you feared for your life or the life of a loved one (e.g., natural disaster, violent crime)
12. None of these

- If YES to any, were any of the above experiences, or other traumatic experiences you have had in your life so frightening, horrible, or upsetting that, during the past 12 months, you…[Yes/No]

i. Had nightmares about it or thought about it when you did not want to?

ii. Tried hard not to think about it or went out of your way to avoid situations that reminded you of it?

iii. Were constantly on guard, watchful, or easily startled?

iv. Felt numb or detached from others, activities, or your surroundings?

*** If any are checked in Q146, display Support Resources** (as a new REDCap page)

The following services are available 24/7 to provide confidential support to individuals in distress, crisis or concerned relationships.

Minnesota Day One® Crisis Line (<https://dayoneservices.org/shelters-support/>)

1-866-223-1111; Text 612-399-9995

Greater Minneapolis Crisis Nursery (<http://crisisnursery.org>)

763-591-0100 (hotline)

Offers free, voluntary services for parents who face barriers such as unemployment, homelessness,

medical or mental health concerns, poverty, domestic violence, or other difficult situations.

Crisis Text Line (<https://www.crisistextline.org/>)

Text HOME to 741741 in the US

National Domestic Violence Hotline (<https://www.thehotline.org/>)

1-800-799-7233

National Suicide Prevention Lifeline (<https://suicidepreventionlifeline.org/>)

1-800-273-8255

National Drug Helpline (<http://drughelpline.org/>)

1-888-633-3239

Substance Abuse and Mental Health Services Administration’s National Helpline (<https://www.samhsa.gov/find-help/national-helpline>)

1-800-662-HELP (4357)

1. In the past 12 months, how often have you been bothered or troubled by …[Radio buttons: Not at all… Very much]

a. Feeling too tired to do things

b. Having trouble going to sleep or staying asleep

c. Feeling unhappy, sad, or depressed

d. Feeling hopeless about the future

e. Feeling nervous or tense

f. Worrying too much about things

1. In the past 12 months …[Radio buttons: Never, once in a while, sometimes, a lot, most of the time, almost all the time]
   1. How frequently have you experienced unfair treatment because you are a woman?
   2. How frequently have you witnessed unfair treatment toward another woman because of her gender?
   3. Have you experienced or witnessed unfair treatment for any other reasons? [Yes/No]

🡪If response is greater than ‘Never’ or ‘Yes’:

For what reason(s)? [Check boxes] race, immigration status, language, gender identity, sexual orientation, weight, social class, other [--> if other, please describe]

*Some women may at times in their life use tobacco, alcohol, prescription drugs and/or other types of drugs. The next set of questions will ask about your use of these products over the last 12 months. Your responses will be kept confidential and names will not be associated with any responses.*

1. In the past 12 months, have you used tobacco or nicotine-containing products (including e-cigarettes)? [Yes/No]

→If YES: What type of tobacco or nicotine-containing products? Check all that apply. [Checkboxes] cigarettes, e-cigarettes, vaping, other

1. In the past 12 months, did you ever drink alcohol? [Yes/No]

🡪 If NO, skip to 152.

🡪 If YES: How often on average did you drink alcohol? By a drink we mean half an ounce of absolute alcohol (e.g. a 12 ounce can or glass of beer or cooler, a 5 ounce glass of wine, or a drink containing 1 shot of liquor). [Drop down] and continue to 151.

1. Every day
2. Nearly everyday
3. 3 or 4 days a week
4. 1 or 2 days a week
5. 3 or 4 days a month
6. About once a month
7. Less than once a month
8. In the past 12 months, what was the largest number of alcoholic drinks you had during a 24-hour period when you drank alcohol?
9. 12 or more drinks
10. 9 to 11 drinks
11. 7 to 8 drinks
12. 5 to 6 drinks
13. 3 to 4 drinks
14. 2 drinks
15. 1 drink
16. In the past 12 months, did you take any of the following medications (at least once a week) for any reason? Check all that apply. [Yes/No]
17. Opioid pain medications such as hydrocodone (Vicodin), oxycodone (Percocet), codeine, or Dilaudid
18. SSRIs (Selective serotonin reuptake inhibitors) such as Sarafem, Zoloft, or Lexapro
19. Other anti-depressants → *If other, please describe: [Textbox]*
20. Benzodiazepines such as Klonopin, Xanax, or Valium
21. Other anti-anxiety medications → *If other, please describe: [Textbox]*
22. Steroids such as prednisone
23. Thyroid medications
24. Anti-nausea/anti-emetic such as Zofran
25. Other → *If other, please describe: [Textbox]*
26. None

- If YES to any, did you ever use [medication] in a way a doctor did not direct you to use it (e.g., used someone else’s prescription; took more than prescribed; took more frequently than prescribed)? [Yes/No]

1. In the past 12 months, did you take any of the following? Check all that apply. [Checkboxes]

- Marijuana
- Heroin/Fentanyl
- Methamphetamine/Psychostimulants
- Prefer not to answer
- None

*Many women experience chronic pain at some point in their lives. By chronic pain we mean pain that lasted more than 3 months (e.g., back pain, migraines, pelvic pain, arthritis).*

1. In the past 12 months, did chronic pain frequently interfere with your mood, activities, relationships, or sleep? [Yes/No]

If NO -> skip to 155

If YES -> continue to 154(a)

(a) Select the best number that describes how, in the past 12 months, chronic pain interfered with your [0 Does not interfere to 5 Completely interferes]:

1. General activity
2. Mood
3. Walking ability
4. Sleep
5. Enjoyment of life

If YES or >0 to any -> 154b

(b) At what age did you first experience this pain?

(c) Did you use any of the following to manage your pain? [Check-boxes]

Over-the-counter medications, prescription medication, physical therapy, meditation/yoga/other holistic therapy (acupuncture or massage), Other [--> if other, please describe]

1. In the past 12 months, how has your sleep quality been overall? [Radio buttons: very good, fairly good, fairly bad, very bad]

# **I. Lifetime health history: Questions for All Women**

1. Have you ever been told by a doctor or other medical provider that you had any of the following? Check all that apply. [Check boxes]

- Type 1 Diabetes
- Type 2 Diabetes
- High blood pressure or heart problems
- Depression
- Anxiety
- Posttraumatic Stress Disorder (PTSD)
- Substance use disorder [excluded if currently pregnant]
- Eating disorder

🡪 What type of eating disorder? Check all that apply. [check boxes] anorexia, bulimia, binge eating disorder, other [if other, please describe___]

- Other mental health condition
- Chronic pain condition (e.g., migraines, arthritis, chronic back pain)
- Autoimmune condition (e.g., Crohn’s disease, lupus)
- Asthma
- Thyroid problems
- Morbid obesity
- Poly-cystic Ovary Syndrome (PCOS)
- Endometriosis
- Cancer

🡪 what type? [open-ended]

- Other medical conditions we should be aware of? [Textbox]
- None

If YES🡪At what age were you first diagnosed with [dx]?

“*Sometimes things about your childhood affect how you grow up. We’d like to know a bit more about you when you were young.”*

1. What is your birthdate [MM/DD/YYYY]?
2. Were you born premature (more than 3 weeks early)? [Yes/No/Don’t know]
3. About how much did you weigh when you were born?

*We know this information may be hard to recall. Please estimate as best you can. If you are not able to answer, please enter 'I don't know.'*

(For reference, the average birth weight for babies is around 7.5 pounds, although between 5.5 and 10 pounds is considered normal. There are 16 ounces in 1 pound.)

1. Pounds [0-14, I don’t know]
2. Ounces [0-15]

- *If* ***I don’t know*** *[pounds] is selected, the following question will display below ounces:*

At birth, would you say you were … [Radio buttons: very small, somewhat small, average size, somewhat large, very large]


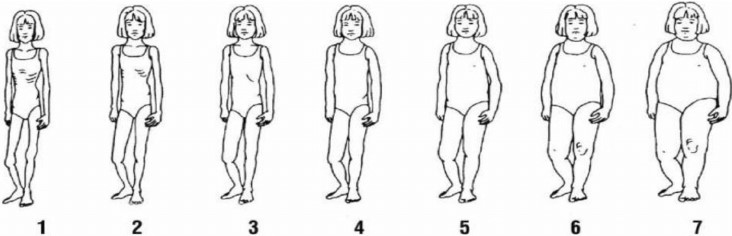


(Collins, 1991)

1. From the above images (will be displayed above the question in REDCap), please choose the picture which looks most like your body shape at age 5 years old: [Radio buttons 1-7]
2. At what age (in years) did you first start menstruating (get your period)?

[Text box w/range 5-20 allowed]

1. About how much did you weigh in pounds at age 18? [Textbox w/#range 50-999 allowed; Checkbox “I don’t know”]
2. Are you planning a pregnancy in the next 2-3 years? [Yes/No/I don’t know]
3. Is there anything else you would like us to know or any feedback you would like to provide? [Textbox]

**[END/SUBMIT]**

Thank you message on last screen.

Participant will also receive a REDCap automatically generated thank you email with details on when to expect gift card, if participant email address provided in survey (or have on file in REDCap).
